# *MN1* Neurodevelopmental Disease-Atypical Phenotype Due to a Novel Frameshift Variant in the *MN1* Gene

**DOI:** 10.3389/fnmol.2021.789778

**Published:** 2021-12-16

**Authors:** Qi Tian, Li Shu, Pu Zhang, Ting Zeng, Yang Cao, Hui Xi, Ying Peng, Yaqin Wang, Xiao Mao, Hua Wang

**Affiliations:** ^1^Department of Medical Genetics, Maternal and Child Health Hospital of Hunan Province, Changsha, China; ^2^National Health Commission Key Laboratory of Birth Defects Research, Prevention and Treatment, Hunan Provincial Maternal and Child Health Care Hospital, Changsha, China; ^3^Department of Obstetrics and Gynecology, Maternal and Child Health Hospital of Hunan Province, Changsha, China; ^4^The Ministry of Education and Science, Maternal and Child Health Hospital of Hunan Province, Changsha, China; ^5^Department of Radiology, Chenzhou First People’s Hospital, Chenzhou, China; ^6^Health Management Center, The Third Xiangya Hospital, Central South University, Changsha, China

**Keywords:** *MN1*, *MN1* C-terminal truncation (MCTT) syndrome, neurodevelopmental outcome, developmental delay, whole-exome sequencing

## Abstract

**Background:** *MN1* C-terminal truncation (MCTT) syndrome is caused by variants in the C-terminal region of *MN1*, which were first described in 2020. The clinical features of MCTT syndrome includes severe neurodevelopmental and brain abnormalities. We reported on a patient who carried the *MN1* variant in the C-terminal region with mild developmental delay and normal brain magnetic resonance image (MRI).

**Methods:** Detailed clinical information was collected in the pedigree. Whole-exome sequencing (WES) accompanied with Sanger sequencing validation were performed. A functional study based on HEK239T cells was performed.

**Results:** A *de novo* heterozygous c.3734delT: p.L1245fs variant was detected. HEK239T cells transinfected with the *de novo* variant showed decreased proliferation, enhanced apoptotic rate, and MN1 nuclear aggregation.

**Conclusion:** Our study expended the clinical and genetic spectrum of MCTT which contributes to the genetic counseling of the *MN1* gene.

## Introduction

*MN1* (MIM 156100) gene was initially reported to be a tumor suppressor gene associated with meningioma and myeloproliferative diseases. Mak et al. (2020) first described the *MN1* C-terminal truncation (MCTT) syndrome as causing craniofacial symptoms and severe neurodevelopmental abnormalities and brain abnormalities (Buijs et al., 1995; Lekanne Deprez et al., 1995). Another research group in the same year reported three probands with *MN1* C-terminal variants who showed consistent clinical features (Vegas et al., 2021).

There is growing evidence of genotype–phenotype correlations of *MN1*-related clinical syndrome. Different from *MN1* C-terminal variants, *MN1* N-terminal variants were reported to cause less severe clinical syndromes. Patients with variants located in the N-terminal region of *MN1* showed speech defects without significant intellectual disability, mild conductive hearing loss, and non-specific facial features (Shu et al., 2021).

Till now, atypical clinical presentations of *MN1*-related clinical syndrome caused by variants in C-terminal region have never been described. In our study, we first presented a *MN1* C-terminal frameshift deletion variant that caused mild global developmental delay, cleft palate, and dysmorphic facial features but with no hearing loss or brain magnetic resonance image (MRI) abnormalities.

## Materials and Methods

### Genetic Investigation

Genomic DNA from peripheral blood leukocytes of the trio were extracted by Qiagen DNA Blood Midi/Mini Kit (Qiagen GmbH, Hilden, Germany). Data were processed preliminarily according to the protocols of whole-exome sequencing (WES) (Ulintz et al., 2019). In detail, DNA was sheared by sonication (Biorupter UCD-200, Diagenode) to approximately 200 bp. DNA fragments were repaired at the end. The sequencing adaptors were used to collect DNA fragments and the fragments (approximately 320 bp) were collected by XP beads. After amplification, the DNA fragments were captured by IDT’s xGen Exome Research Panel (Integrated DNA Technologies, San Diego, CA, United States) according to the protocol. The products were eluted and collected. DNA was then amplified and purified by PCR. The enrichment of libraries was tested by qPCR, and size distribution and concentration were determined by Agilent Bioanalyzer 2100 (Agilent Technologies, Santa Clara, CA, United States). To sequence the genomic DNA of the family, WES was performed on the Illumina HiSeq 2500 system with an average coverage depth of 100× of the variants. Raw image files were processed using CASAVA v1.82 for base calling and raw data generating (Markus et al., 2020). Variants were then annotated using ANNOVAR (Wang et al., 2010).

The variants were initially filtered following HGMD and ACMG guidelines. Disease-causing mutations (DMs) and probable/possible pathological mutation (DM) in the HGMD database (Prof. version 2019.1), and pathogenic (P) and likely pathogenic (LP) variants were interpreted by ACMG guidelines. The variants were then filtered according to allele frequency, variant type, and mode of inheritance. Variants with minor allele frequencies (MAFs) <0.1%, variant depth of coverage ≥20, and alteration base depth of coverage ≥4 were chosen for further analyses. The remaining variants were further filtered according to variant type and inheritance model of the associated disease. Sanger sequencing was performed on the DNA of the proband’s parents to validate the mutation found in WES.

### MN1 Subcellular Localization and Aggregation in HEK293T Cells

To create N-terminal GFP-fused human *MN1* expression vector, the *MN1* (GenBank: NM_002430.3) open-reading frame (ORF) was incorporated into DEST53 *via* the Gateway cloning system (Thermo Fisher). The mutant ORF and *MN1* was amplified with a human cDNA library (Clontech). The mutant *MN1* (M-MN1) was created by a KOD-plus-Mutagenesis Kit (TOYOBO). ViaFect Transfection Reagent (Promega) was used to transfect construct (500 ng each) into HEK239T cells. After 48 h of transfection, the cells were fixed with 2% paraformaldehyde, washed with PBS, stained with DAPI (Vector Laboratories), and then mounted onto slides. The sub-cellular localization and aggregation of MN1/M-MN1 were observed under confocal microscopy.

### Cell Proliferation and Apoptosis Assay

Cell Proliferation Assay was carried out with Cell Counting Kit-8 (CCK-8, Dojindo Laboratories, Kumamoto, Japan) according to the manufacturer’s protocol (Vegas et al., 2021). Cell Apoptosis Assay Kit (Solarbio, CA1020) was used to detect apoptotic rate (Xiao et al., 2019).

## Results

### Case Description

**FIGURE 1:**
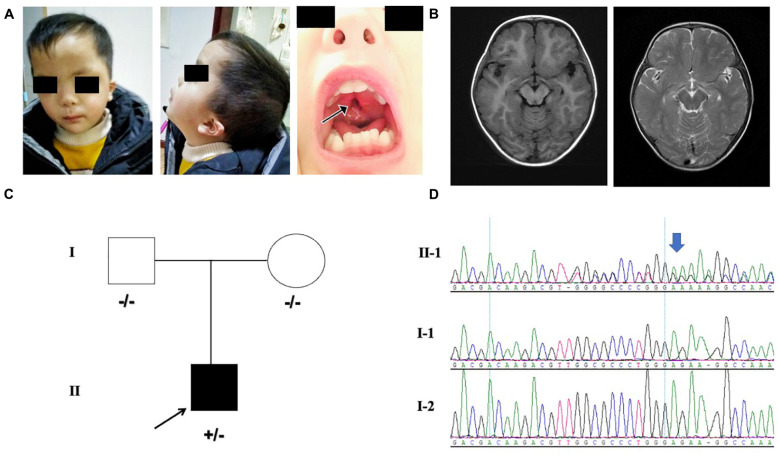
**(A)** Facial features of the proband. The arrows point to the cleft palate. **(B)** MRI indicating the normal brain tissue and the protruding occipital bone (image on the left is axial T1-weighted; image on the right is axial T2-weighted) of the proband at 18-months-old. **(C)** Pedigree with *MN1* variant. Individuals with heterozygous variants are indicated by plus/minus (+/–) symbols and individuals without the variant are labeled as minus/minus (–/–) symbols. **(D)** Sanger sequencing results of *MN1* frameshift variant in family members is presented on the right.

Our patient was the first child born to the non-consanguineous Chinese parents. The proband was a 3 year and 5-month-old male born at full term from a normal pregnancy. The birth weight was 2800 g and Apgar score was 9/10. The patient presented difficulties in breast feeding. He lifted his head at 7 months old and could sit with support at the age of 8 months. He stared to walk when he was 1 year and 7 months old. He had no seizures and language development delay was observed. Physical examination showed that he had facial dysmorphism, hypertelorism, auricle deformation, upper palate cleft, plagiocephaly, protruding occipital bone, and hypotonia (Figure 1A). Behavioral observation audiometry and auditory brainstem response were normal. The brain MRI was normal (Figure 1B). The developmental milestones were presented in weight/length-for-age percentiles (Supplementary Figure 1). A *de novo MN1* gene frameshift variant NM_002430.2: c.3734delT: p.L1245fs (chr22:28192798-28192798) was identified and proved by sanger sequencing in the pedigree (Figures 1C,D). The table showing the follow-up timeline is available in Supplementary Table 1.

### Functional Study for *MN1* Variant in HEK239T Cells

**FIGURE 2:**
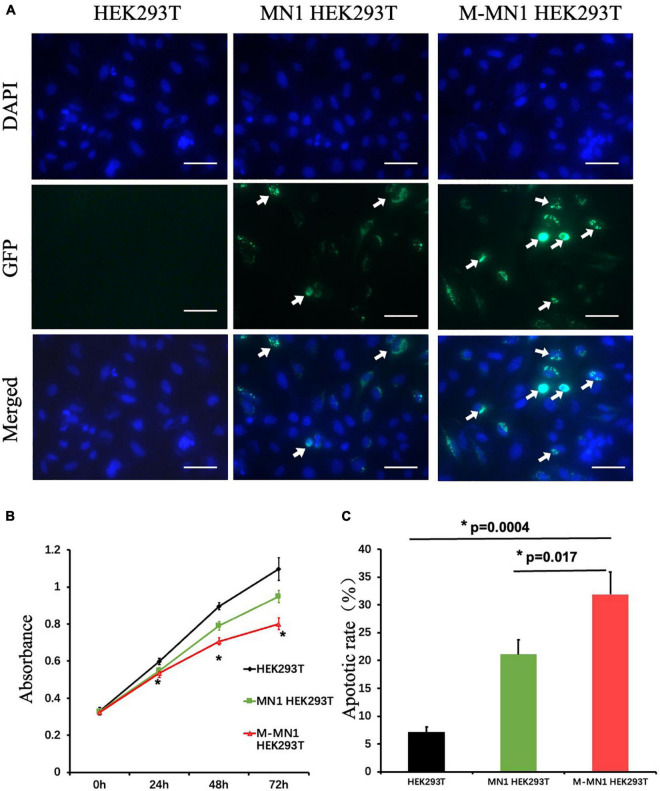
**(A)** Immunofluorescence of GFP-fused MN1 and M-MN1 showing the subcellular localization and aggregation of MN1 proteins in HEK239T cells. The arrows pointed out the represented MN1 fluorescence. Scale bars represent 50 mm. **(B)** Cell proliferation at 0, 24, 48, and 72 h after transinfection. The cell numbers indicated by the absorbance at 450 nm were significantly lower in transinfected groups (*t*-test, *p* = 0.017). And the M-MN1 group had the lowest absorbance reading across all time points; at 48 and 72 h the readings of M-MN1 group were significantly lower compared to MN1 group (*t*-test, *p* = 0.009, 0.004, respectively). **(C)** Cell apoptotic rate was significantly higher in M-MN1 group compared with HEK239T and MN1 group. *p ≤ 0.05.

GFP-fused wild-type and mutated-MN1 proteins were expressed in HEK239T cells. Both wild-type MN1 and M-MN1 were found to be aggregated and localized in the nuclear of HEK239T cells (Figure 2A). Intensity of M-MN1 aggregates were significantly higher than the wild-type MN1 (*t*-test, *p* = 0.017) (Figure 2B). Cell apoptotic rate were statistically higher in M-MN1 group compared with HEK239T and wild-type group (*t*-test, *p* = 0.009, 0.004, respectively) (Figure 2C).

## Discussion

The clinical characteristics of MCTT syndrome included craniofacial features, hearing loss, severe neurodevelopmental abnormalities, and abnormal brain MRI (Vegas et al., 2021). In our study, we identified a *de novo* frameshift deletion variant located in C-terminal of *MN1* gene in a pedigree. Our proband presented a few atypical clinical manifestations different from the reported ones (Mak et al., 2020; Miyake et al., 2020). Our patients did not show cranial shape defects, hearing loss, or brain MRI abnormalities (Table 1). Our patient provided evidence for *MN1* MCTT syndrome and the different clinical features of our patient may help to refine the clinical spectrum of *MN1* C-terminal variants’ related syndromes.

**TABLE 1:** The clinical comparison of our patient and the patients reported previously.

Dysmorphisms	The reported patients	Our patient
Cranial shape defects	+	+
Typical facial defects	+	+
Hearing loss	+	−
Developmental delay	+	+
Feeding difficulty	+	+
Hypotonia	+	+
Brain MRI abnormality	+	−

*MN1* C-terminal heterozygous variants exert a dominant-negative or gain-of-function effect on the MN1 protein (Mak et al., 2020). The variant led to increased protein MN1 stability and enhanced MN1 nuclear aggregation, which were related to the MCTT syndrome (Miyake et al., 2020). In our study, the variant was tested by functional study and the abnormal cellular functions were detected in M-MN1 group. Combining the functional studies with the genetic findings, we proved that our MN1 variant was the cause of the diseases. Further research on how different *MN1* variants lead to various clinical manifestations is needed.

Some research has revealed the molecular functions of MN1 protein. *MN1* encodes a developmentally expressed transcriptional co-regulator (Liu et al., 2008). MN1 protein may act as a transcriptional cofactor and the mutant protein could impair downstream binding transcription factors, such as Cbf-β and Runx2 (Meester-Smoor et al., 2005; Miyake et al., 2020). The clinical heterogeneities of *MN1* C-terminal variants may be due to the regulation of various corresponding downstream target genes (Lai et al., 2014). Our present study contributes to expanding the genetic and clinical spectrum of *MN1* and aid in precise genetic counseling in the future.

## Data Availability Statement

The data that support the findings of this study are available from the corresponding author (XM), upon reasonable request. Requests to access these datasets should be directed to XM (gbtechies@outlook.com).

## Ethics Statement

The studies involving human participants were reviewed and approved by the Ethics Committee of the Maternal and Child Health Hospital of Hunan Province (2020-S003). Written informed consent to participate in this study was provided by the participants’ legal guardian/next of kin. Written informed consent was obtained from the minor(s)’ legal guardian/next of kin for the publication of any potentially identifiable images or data included in this article.

## Author Contributions

XM, HW, and YW designed the research. QT, LS, and TZ interpreted the data and wrote the manuscript. QT, LS, PZ, YC, HX, and YP did the follow-up study and collected, evaluated the clinical, and genetic evidence. TZ revised the manuscript. All authors read and approved the final manuscript.

## Conflict of Interest

The authors declare that the research was conducted in the absence of any commercial or financial relationships that could be construed as a potential conflict of interest.

## Publisher’s Note

All claims expressed in this article are solely those of the authors and do not necessarily represent those of their affiliated organizations, or those of the publisher, the editors and the reviewers. Any product that may be evaluated in this article, or claim that may be made by its manufacturer, is not guaranteed or endorsed by the publisher.
