# Supplementary material for: MN1 Neurodevelopmental Disease-Atypical Phenotype Due to a Novel Frameshift Variant in the MN1 Gene
Source: Front Mol Neurosci. 2021 Dec 16;14:789778. doi: 10.3389/fnmol.2021.789778 (PMC8716923; doi:10.3389/fnmol.2021.789778)
Supplement: Supplementary file 1 [file Data_Sheet_1.docx]

Supplementary Material

Figure 1 The developmental milestones


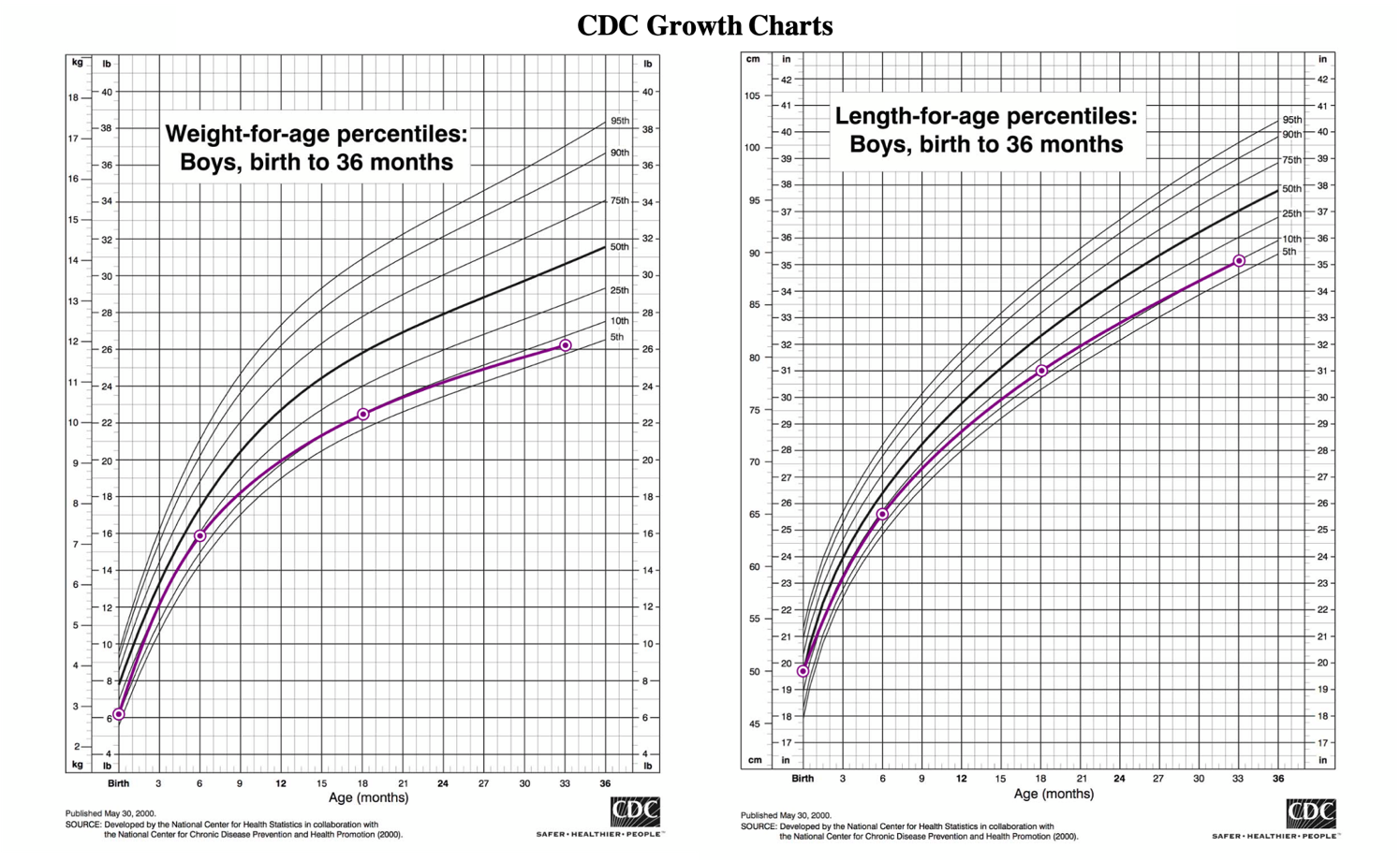


Figure Legend

**Figure 1** The developmental milestones were measured and presented in weight-for-age percentiles (on the left) and length-for-age percentiles (on the right), the purple dots represented the age(months) of each evaluation and the curve of development (purple lines) were generated. As shown in the charts, the weight and length-age percentiles were around 10^th^ percentiles of the population. The source of the charts: The National Center for Health Statistics in collaboration with the National center for Chronic Disease Prevention and Health Promotion (2000).

Table 1 Follow up timeline of the proband

| **Age of visits**  **(months)** | **Summaries from Initial**  **and Follow-up Visits** | **Diagnostic Testing** | **Interventions** |
| --- | --- | --- | --- |
| 0 | Uneventful birth with normal Apgar score and hearing screening | Initial evaluation | Basic testing and vaccination |
| 7 | Development evaluation and screening for genetic diseases | Genetic testing | Diagnoses and instructions for feeding |
| 18 | Development evaluation  and brain assessment | Brain MRI | N/A |
| 36 | Development and hearing testing | Behavioural observation audiometry/ Auditory brainstem response tests | Rehabilitation exercise |
